# Childhood neglect is associated with alterations in neural prediction error signaling and the response to novelty

**DOI:** 10.1017/S0033291724002411

**Published:** 2024-10

**Authors:** Joseph Aloi, Kathleen I. Crum, Karina S. Blair, Ru Zhang, Johannah Bashford-Largo, Sahil Bajaj, Soonjo Hwang, Bruno B. Averbeck, Nim Tottenham, Matthew Dobbertin, R. James R. Blair

**Affiliations:** 1 Department of Psychiatry, Indiana University School of Medicine, Indianapolis, IN, USA; 2 Adolescent Behavioral Health Research Program, Indiana University School of Medicine, Indianapolis, IN, USA; 3 Department of Neuroscience, Medical University of South Carolina, Charleston, SC, USA; 4 Center for Neurobehavioral Research, Boys Town National Research Hospital, Boys Town, NE, USA; 5 Stevens Neuroimaging and Informatics Institute, Keck School of Medicine, University of Southern California, Los Angeles, CA, USA; 6 Child and Family Translational Research Center, Boys Town National Research Hospital, Boys Town, NE, USA; 7 Department of Cancer Systems Imaging, Division of Diagnostic Imaging, The University of Texas MD Anderson Cancer Center, Houston, TX, USA; 8 Department of Psychiatry, University of Nebraska Medical Center, Omaha, NE, USA; 9 Section on Learning and Decision Making, Laboratory of Neuropsychology, National Institute of Mental Health, Bethesda, MD, USA; 10 Department of Psychology, Columbia University, New York, NY, USA; 11 Child and Adolescent Mental Health Centre, Mental Health Services, Capital Region of Denmark, Copenhagen, Denmark, USA

**Keywords:** early life stress, fMRI, reinforcement learning

## Abstract

**Background:**

One in eight children experience early life stress (ELS), which increases risk for psychopathology. ELS, particularly neglect, has been associated with reduced responsivity to reward. However, little work has investigated the computational specifics of this disrupted reward response – particularly with respect to the neural response to Reward Prediction Errors (RPE) – a critical signal for successful instrumental learning – and the extent to which they are augmented to novel stimuli. The goal of the current study was to investigate the associations of abuse and neglect, and neural representation of RPE to novel and non-novel stimuli.

**Methods:**

One hundred and seventy-eight participants (aged 10–18, *M* = 14.9, s.d. = 2.38) engaged in the Novelty task while undergoing functional magnetic resonance imaging. In this task, participants learn to choose novel or non-novel stimuli to win monetary rewards varying from $0 to $0.30 per trial. Levels of abuse and neglect were measured using the Childhood Trauma Questionnaire.

**Results:**

Adolescents exposed to high levels of neglect showed reduced RPE-modulated blood oxygenation level dependent response within medial and lateral frontal cortices particularly when exploring novel stimuli (*p* < 0.05, corrected for multiple comparisons) relative to adolescents exposed to lower levels of neglect.

**Conclusions:**

These data expand on previous work by indicating that neglect, but not abuse, is associated with impairments in neural RPE representation within medial and lateral frontal cortices. However, there was no association between neglect and behavioral impairments on the Novelty task, suggesting that these neural differences do not necessarily translate into behavioral differences within the context of the Novelty task.

## Introduction

One in eight children in the United States experience early life stress (ELS) as maltreatment by the age of eighteen (Wildeman et al., 2014). Childhood ELS exposure increases the risk for psychopathology during adolescence and adulthood, including anxiety, depression, conduct disorder, and substance use disorders (Carliner, Gary, McLaughlin, & Keyes, 2017; Carliner et al., 2016; McLaughlin, DeCross, Jovanovic, & Tottenham, 2019). This increased risk for psychopathology likely reflects the adverse neurodevelopmental impacts of ELS (McLaughlin et al., 2019).

ELS exposure is associated with impacts in at least three neural systems: threat sensitivity (McLaughlin, Peverill, Gold, Alves, & Sheridan, 2015; Pine et al., 2005; Tottenham et al., 2011), executive function (Harms, Shannon Bowen, Hanson, & Pollak, 2018), and reinforcement learning (Birn, Roeber, & Pollak, 2017; Gerin et al., 2017). Animal work indicates that ELS exposure disrupts the development of frontostriatal neuro-circuitry critical for reward processing (Stedenfeld et al., 2011). Human neuroimaging work has shown that ELS exposure is associated with reduced neural responsivity to rewards within striatum and prefrontal cortex (Hanson, Hariri, & Williamson, 2015; Mehta et al., 2010). These systems are critical for reinforcement learning-the process by which an organism learns the values of actions or choices in order to achieve higher value states (Averbeck & O'Doherty, 2022). Reinforcement learning (RL) relies on successful neuro-computation of two values: expected value (EV) and reward prediction error (RPE). EV is the subjective value associated with a state or action and is learned through experience with the state or action. RPE is the difference between the actual value of the feedback received following a state transition or action, and triggers a revision of EV (Rescorla & Wagner, 1972). Thus, if the actual reward received is greater than the EV, the EV will be increased, such that next time that action is selected or the state is experienced, the EV will be larger. Prior work has shown that in adolescent girls with histories of being victims of assault (Cisler et al., 2019) or sexual abuse (Letkiewicz, Cochran, & Cisler, 2022), there are impairments in neural representation of RPE. In short, individuals with histories of ELS show reduced striatal responsiveness to reward (Dillon et al., 2009; Mehta et al., 2010) and disrupted RPE signaling (Cisler et al., 2019; Letkiewicz et al., 2022).

Striatal RPE representation is enhanced in organisms while exploring novel stimuli (Costa, Mitz, & Averbeck, 2019; Wittmann, Daw, Seymour, & Dolan, 2008). This RPE enhancement is thought to underpin the exploration of novel stimuli (Wittmann et al., 2008). Novelty-seeking refers to an individual's tendency to explore unfamiliar stimuli at the expense of exploiting the known properties of familiar stimuli to attain the highest-valued states or actions in the future (Cloninger, Svrakic, & Przybeck, 1993). Neuroimaging work has associated trait novelty-seeking with reduced midbrain dopaminergic signaling (Zald et al., 2008) and novelty signaling with enhancement of striatal RPE signals (Wang et al., 2015; Wittmann et al., 2008). Few studies to date have examined group differences in RPE signaling based on ELS exposure (Cisler et al., 2019; Letkiewicz et al., 2022; Palacios-Barrios et al., 2021), however, none examined augmentation of RPE signaling when exploring novel stimuli.

Group differences between different forms of ELS on RL has also received relatively little attention. Prior work has grouped together different forms of ELS (Birn et al., 2017; Gerin et al., 2017) or investigated one specific form of ELS, such as emotional neglect (Hanson et al., 2015) or institutionalization (Mehta et al., 2010). Different forms of childhood ELS may have distinct neurodevelopmental consequences, although children who experience one form of ELS often experience multiple forms of ELS (McLaughlin & Sheridan, 2016). Specifically, it has been suggested that children exposed to threat-related ELS (e.g. abuse) show abnormalities in fear processing while children exposed to deprivation-related ELS (e.g. neglect) show impairments in RL (McLaughlin & Sheridan, 2016). Indeed, behavioral data have shown that deprivation-related ELS is associated with reduced reward learning as indexed behaviorally (Sheridan et al., 2018). However, only one study to date has evaluated the distinct associations between abuse and neglect on neural dysfunction underlying instrumental learning. Our prior work has shown that in a passive avoidance learning task, level of neglect, but not level of abuse, is associated with reduced differential reward-punishment responsiveness (Blair et al., 2022a). However, this study did not investigate neuro-computational impairments in RPE signaling as a function of neglect.

The current study aimed to investigate group differences in different types of maltreatment (abuse and neglect) in neural RPE representation, and whether this is augmented for novel stimuli, during instrumental learning. In particular, the current study aimed to examine this issue in the context of an instrumental learning paradigm where participants must decide whether to explore novel stimuli with relatively unknown EVs or exploit familiar stimuli with relatively more known EVs (Costa et al., 2019). It has been considered important to examine RL as the information gained may provide insight into neural mechanisms underlying behavior change that may inform intervention strategies (Brown et al., 2021). Our prior work (Blair et al., 2022a, 2022b) showed that individuals with greater levels of neglect showed reduced reward responsivity during an instrumental learning task. Therefore, we hypothesized that individuals with significant histories of emotional or physical neglect would show reduced RPE-modulated blood oxygenation level dependent (BOLD) response within striatum and medial prefrontal cortex. Moreover, if neglect is associated with an interruption in neural RPE signaling, then these impairments should be particularly pronounced when exploring novel stimuli.

## Methods

### Participants and recruitment

Participants aged 10–18 years old were recruited either from a residential youth care facility (*n* = 70) or the surrounding community (*n* = 108), and combined to create a sample (*N* = 178) from which abuse and/or neglect could be assessed. Participants were recruited for a broader study investigating neural correlates in adolescents with behavioral and emotional problems. See Table 1 and online Supplemental material for further details regarding recruitment.

**Table 1.** Demographic information and clinical variables

	Lo Abuse/Neglect	Hi abuse only	Hi neglect only	Hi abuse/neglect	F/chi-square	Comparison
*N*	78	21	27	52		
% from residential facility	0%	52.3%	51.9%	85.5%		
EA ≥ 9	0	14	0	46		
PA ≥ 8	0	9	0	33		
SA ≥ 6	0	2	0	20		
PN ≥ 10	0	0	17	49		
EN ≥ 8	0	0	15	32		
Age (s.e.)	14.0 (0.25)	16.2 (0.48)	14.3 (0.42)	16.1 (0.31)	206.20* 0.28 0.14	Hi abuse > Lo abuse* Main effect of neglect Abuse-by-neglect interaction
% Male	56.4%	57.1%	63.0%	46.2%	2.41	
IQ (s.d.)	107.4 (13.53)	103.2 (12.22)	103.8 (12.63)	99.0 (11.16)	239.8* 181.59* 0.02	Hi abuse > Lo abuse* Hi neglect > Lo neglect* Abuse-by-neglect interaction
CTQ EA	5.5 (0.85)	9.0 (2.53)	6.1 (1.14)	13.7 (4.19)	165.59* 37.06* 22.91*	Hi abuse > Lo abuse* Hi neglect > Lo neglect* Hi abuse/neglect > abuse only > neglect only = Lo abuse/neglect*
CTQ PA	5.1 (0.27)	6.9 (1.45)	5.4 (0.70)	9.4 (3.78)	62.67* 15.20* 7.94*	Hi abuse > Lo abuse* Hi neglect > Lo neglect* Hi abuse/neglect > abuse only > neglect only = Lo abuse/neglect*
CTQ SA	5.0 (0)	6.5 (4.88)	5.0 (0)	8.0 (5.23)	16.11* 1.90 1.90	Hi abuse > Lo abuse* Main effect of neglect Abuse-by-neglect interaction
CTQ EN	5.3 (0.74)	6.5 (1.37)	10.6 (4.15)	13.6 (3.69)	20.46* 187.55* 3.77	Hi abuse > Lo abuse* Hi neglect > Lo neglect* Abuse-by-neglect interaction
CTQ PN	5.3 (0.53)	5.5 (0.75)	7.9 (2.83)	9.1 (3.81)	2.6858.98* 1.34	Main effect of abuseHi neglect > Lo neglect* Abuse-by-neglect interaction
CTQ total	26.2 (1.68)	34.3 (6.15)	35.1 (5.51)	53.7 (12.85)	104.98* 116.92* 15.89*	Hi abuse > Lo abuse* Hi neglect > Lo neglect* Hi abuse/neglect > abuse only = neglect only > Lo abuse/neglect*
% ADHD	0%	47.6%	59.3%	59.6%	0.96	
% CD	0%	38.1%	40.7%	63.5%	5.74	
% GAD	0%	28.6%	14.8%	48.1%	9.13^a^*	Hi abuse/neglect> neglect only^a^*
% MDD	0%	9.5%	7.4%	26.9%	5.88	
% PTSD	0%	4.8%	0%	28.8%	13.50^a^*	Hi abuse/neglect > abuse only = neglect only^a^*

*N* *=* 178.

a Indicates comparisons between abuse only, neglect only, and co-morbid abuse/neglect groups.

*Indicates significant differences at *p* < 0.05.

Parental consent and youth assent was obtained from all participants. Fifty three participants were excluded from the sample based on excessive movement, fMRI artifact, or missing questionnaire data. This resulted in a final sample of 178 adolescents; average age = 14.9, average IQ = 103.9, 97 males (see ELS assessments section below for details on how groups were created). Demographic and maltreatment data for the excluded participants are reported in the online Supplemental material.

### Exclusion criteria

See online Supplemental material for exclusion criteria.

### Measures

#### Els assessments

Extent of abuse/neglect was determined via the Child Trauma Questionnaire (CTQ) (Bernstein, Ahluvalia, Pogge, & Handelsman, 1997) and validated with clinical records. Cut-off scores for both abuse (Emotional Abuse >8, Physical Abuse >7, and/or Sexual Abuse >5) and neglect (emotional neglect > 9 and/or physical neglect > 7) are specified in the CTQ manual (Bernstein et al., 1997). Participants were divided into four groups according to whether they had been exposed to high: (i) abuse and neglect (individuals above cutoff for at least one form of abuse *and* one form of neglect); (ii) abuse but not neglect (individuals above cutoff for at least one form of abuse *but* no forms of neglect); (iii) neglect but not abuse (individuals above cut-off for at least one form of neglect *but* no forms of abuse); and (iv) healthy control (comparison individuals below cut-off for all forms of abuse and neglect and without any psychopathology). Individuals from the three ELS groups came from both the residential treatment facility and the community, whereas individuals in the healthy control group came exclusively from the community.

#### Novelty task

**Figure 1.**
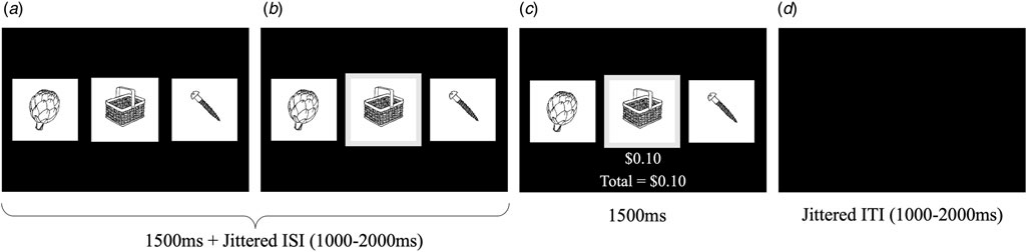
Diagram of the Novelty Task. The Novelty Task is a three-armed bandit task where: (A) Three stimuli are presented at the beginning of each trial. (B) Participant chooses one of the three stimuli. (C) Participant receives feedback ($0–$0.30) based on the stimulus chosen on that trial. (D) Intertrial interval between trials.

The Novelty Task (Fig. 1) (Djamshidian, O; ullivan, Wittmann, Lees, & Averbeck, 2011) is a three-armed bandit task where participants are presented with three stimuli on each trial. The specific location (left, middle, right) of each stimulus was randomized per trial. Each stimulus was presented for a series of 5–9 consecutive trials before being replaced with a novel stimulus. Participants encountered a total of 40 novel stimuli during the task. Participants are informed prior to the task that each picture has been assigned a unique probability of winning a unique value between $0.00–$0.30 and that they would receive 10% of their overall winnings at the conclusion of the task. For further details, see online Supplemental Material.

### Computational modeling

EV and RPE were modeled using techniques developed by Averbeck and colleagues (Djamshidian et al., 2011). Using this procedure, an RL model was fit to the choice data of participants to determine two parameters: learning rate (*α*) and inverse temperature (*β*). Based on each participant's individual behavioral data, a learning curve was modeled establishing EVs and RPEs for each stimulus on each trial. RPE was calculated as the difference between the feedback (F) and EV for the chosen stimulus with the formula:
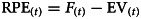


EV was updated for the chosen stimulus for each trial with the following formula:
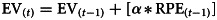


Based on a broad sample of 290 individuals who completed the novelty task (including current participants as well as participants who completed a pilot study and were held out of the current study) (Aloi et al., 2021), a learning rate of *α* = 0.692 was established. We used this fixed learning rate for several reasons. First, our goal was to use a population learning rate for this task and to focus on group differences rather than individual differences. Second, this approach minimizes collinearity in the model, as the behavioral learning rate and prediction error signal at least partly reflect the same computational process.

A logistic regression procedure was performed to calculate EV of novel stimuli. Briefly, the value of a novel stimulus was established as the value at which there was a 0.5 probability that the participant would choose the novel stimulus over the best (highest EV) alternative stimulus on the second trial after a novel stimulus was presented, as participants most frequently chose the novel stimulus on the second trial after a novel stimulus was presented. Trials on which participants selected the novel stimulus on the second trial after a novel stimulus was presented are termed ‘Explore’ trials while all other trials are termed ‘Non-Explore’ trials. Based on a sample of 290 participants who completed the novelty task, the average EV of the novel stimulus on Explore trials was 0.216, so novel stimuli were assigned an initial EV of 0.216. For further details on modeling approaches, see online Supplemental Material.

### Functional MRI parameters and individual-level analysis

For Functional MRI parameters and individual-level analysis, see online Supplemental Material.

### Statistical analysis plan

#### Demographics

To investigate group differences in IQ and age, we conducted two (Abuse: High v. Low) × 2 (Neglect: High v. Low) ANOVAs on IQ and age. We also calculated odds ratios to assess whether gender was associated with abuse or neglect.

#### Clinical data

To investigate relationships with common psychopathologies (ADHD, CD, MDD, GAD, PTSD) or gender, we ran a series of chi-square tests to test whether there were significant group differences in incidence rates of these psychopathologies. The group of typically developing participants were excluded from these Chi-square tests.

#### Behavioral and motion data

To investigate group differences in behavior on the Novelty task, we conducted two 2 (Abuse: High *v.* Low) × 2 (Neglect: High *v.* Low) ANOVAs on proportion of best non-novel stimuli chosen and on novelty propensity (NP). Second, we conducted a 2 (Abuse: High *v.* Low) × 2 (Neglect: High *v.* Low) ANOVA on average motion per TR.

#### BOLD response fMRI data

The group level analysis was an ANCOVA conducted using AFNI's 3dMVM tool. We investigated group differences in BOLD response modulated by RPE via a 2 (Abuse: High *v.* Low) × 2 (Neglect: High *v.* Low) × 2 (Explore: Explore, Non-Explore) ANCOVA with NP as a covariate. This analysis examined main and interactive effects between abuse, neglect, and NP on RPE-modulated BOLD response. The between-subjects variables were Abuse, Neglect, and NP. The within subject variable was Explore. 3dMVM automatically calculates all main effects and higher-order interaction effects for all between subjects and within subject variables. Post hoc analyses were conducted on the percent signal change taken from all significant voxels within each functional ROI generated by AFNI (i.e. all clusters that survive the above threshold of initial *p* < 0.001 and cluster size *k* ≥ 17 voxels) to examine significant main effects and interactions with planned post hoc tests within SPSS 28.0. Within SPSS, we ran a 2 (Abuse: High *v.* Low) × 2 (Neglect: High *v.* Low) × 2 (Explore: Explore, Non-Explore) ANCOVA with NP as a covariate for F-statistics reported in Table 2. For clusters showing a main effect, we conducted post-hoc two-sample *t* tests on the average extracted RPE-modulated BOLD response in each cluster. For clusters showing interaction effects, we conducted two-sample *t* tests on the extracted RPE-modulated BOLD response in explore and non-explore trials separately. Effect sizes (partial eta-squared) for all clusters are reported to facilitate meta-analyses. For details regarding multiple comparison correction, see online Supplemental material.

**Table 2.** Brain regions demonstrating significant neglect and neglect-by-explore effects

Coordinates of peak activation^a^								
Region^b^	Hemisphere	BA	*x*	*y*	*z*	*F*(1174)	Partial *η*^2^	Voxels
*Main effect of neglect*								
rmPFC	R	9	8	44	29	17.48	0.093	23
ACC/vmPFC	R	32	5	41	11	19.95	0.104	20
dlPFC	R	10	20	59	26	19.89	0.105	30
*Neglect-by-explore*								
ACC/vmPFC/rmPFC	R	9/10/32	5	44	14	20.20	0.106	31
dlPFC	R	10	29	59	2	25.59	0.131	37
Postcentral gyrus	L	3	−22	−31	50	18.72	0.099	21
Superior frontal gyrus	R	6	20	14	56	17.53	0.093	18
*Abuse-by-neglect-by-NP-by-explore*								
Cuneus	R	19	29	−82	26	19.20	0.101	20

*Note*:

a Based on the Tournoux and Talairach standard brain template. BA, Brodmann's area.

b According to the Talairach Daemon Atlas (http://www.nitrc.org/projects/tal-daemon/).

## Results

### Demographics

The average IQ for the final sample of *N* = 178 participants was 103.9 (s.d. = 12.99) and the average age was 14.9 (s.d. = 2.38). Two 2 (Abuse: High *v.* Low) × 2 (Neglect: High *v.* Low) ANOVAs were conducted on the IQ and age data respectively. These revealed, for IQ, a significant main effect of both abuse [*F*(1173) = 239.8, *p* < 0.05] and neglect [*F*(1173) = 181.6, *p* < 0.05]. IQ was lower in individuals exposed to high levels of abuse (*M* = 100.2, s.d. = 11.56) than in individuals exposed to lower levels of abuse (*M* = 106.5, s.d. = 13.34) and in individuals with high levels of neglect (*M* = 100.6, s.d. = 11.83) than in individuals exposed to lower levels of neglect (106.5, s.d. = 13.31). For age, there was a significant main effect of abuse [*F*(1173) = 206.2, *p* < 0.05]; individuals exposed to high levels of abuse were younger (*M* = 14.1, s.d. = 2.52) than individuals exposed to lower levels of abuse (*M* = 16.1, s.d. = 1.52). There were no other differences (main or interaction effects) between groups on IQ or age.

The sample consisted of 97 males and 81 females. Gender was not significantly associated with neglect (Odds Ratio (OR) = 0.96, 95% CI 0.49–1.88) or abuse (OR = 0.714, 95% CI 0.36–1.40). Moreover, within the three groups with clinically significant abuse and/or neglect, chi-square testing showed that there were no significant differences in gender between these groups (χ^2^ = 2.20, *p* > 0.05).

### Clinical data

Within the three groups with clinically significant abuse and/or neglect, chi-square tests showed that there were significant differences in proportions of individuals with Generalized Anxiety Disorder (GAD) (χ^2^ = 9.13, *p* < 0.05) and Posttraumatic Stress Disorder (PTSD) (χ^2^ = 13.50, *p* < 0.05). These group differences were primarily driven by the group exposed to clinically significant abuse & neglect presenting with higher levels of both GAD and PTSD (48.1% & 28.8%, respectively) relative to the groups of individuals exposed to high abuse only (28.6%/4.8%, respectively) and high neglect only (14.8%/0%, respectively). There were no group differences in proportions of individuals with Major Depressive Disorder (MDD), Conduct Disorder (CD), or Attention Deficit/Hyperactivity Disorder (ADHD) (χ^2^'s < 5.88, *p*'s > 0.05).

### Behavioral data

There was no main effect of neglect, main effect of abuse, or neglect-by-abuse interaction effect on response time, amount of money won, NP, learning rate, or proportion of best non-novel stimuli chosen (*F*s = 0–30.00, *p*s > 0.05). The average response time on non-explore trials was 650.4 ms (s.d. = 87.90 ms) and the average response time on explore trials was 646.0 ms (s.d. = 98.30 ms). The average amount of money won was $48.26 (s.d. = $6.418).

### fMRI results

#### Movement data

There were no main effects of neglect, abuse, or neglect-by-abuse interactions on average motion per TR [*F*s = 1.75–7.47, *p*s > 0.05].

#### BOLD response data

A 2 (Abuse: High, Low) × 2 (Neglect: High, Low) × 2 (Explore: Explore, Non-Explore) ANOVA was conducted on BOLD Response Data modulated by RPE. This analysis revealed the following significant effects:

*Main effect of neglect:* There was a significant main effect of neglect on RPE-modulated BOLD response within rostromedial prefrontal cortex, ventromedial prefrontal/anterior cingulate cortex, and dorsolateral prefrontal cortex. Individuals with significant histories of neglect showed reduced RPE-modulated BOLD response within all brain regions relative to individuals without significant histories of neglect (*t*s = −4.12–3.86, *p*s < 0.001). Note though that this main effect of neglect within these regions primarily reflected the high significance of the Neglect-by-Explore interaction; there was no significant effect of Neglect for the non-Explore trials. See Table 2 for further details.

**Figure 2.**
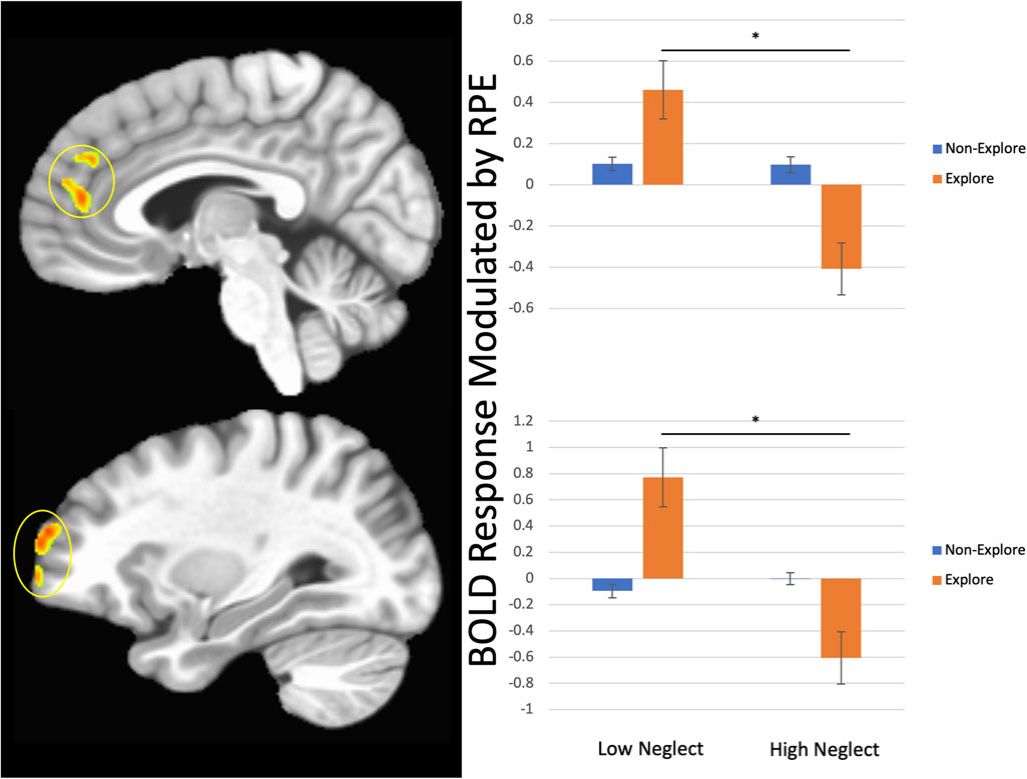
Neglect-by-explore interaction effects on BOLD response modulated by RPE within ACC/vmPFC/rmPFC (top panel) and dorsolateral prefrontal cortex (bottom panel). Within all brain regions, individuals who had experienced high levels of neglect showed reduced RPE-modulated BOLD response on explore trials relative to non-explore trials.

*Neglect-by-explore interaction:* There was a significant neglect group-by-explore interaction on RPE-modulated BOLD response within rostro/ventromedial prefrontal cortex/anterior cingulate cortex, dorsolateral prefrontal cortex, postcentral gyrus, and superior frontal gyrus. Individuals with significant histories of neglect showed reduced RPE-modulated BOLD responses during explore trials compared to individuals without significant histories of neglect (*t*s = −4.48–3.95, *p*s < 0.001). See Fig. 2 and Table 2 for further details.

*Abuse-by-NP-by-explore interaction:* There was a significant abuse-by-NP-by-explore interaction within cuneus. See Table 2 for further details.

There were no significant main effects of abuse or abuse-by-explore interaction effects on RPE-modulated BOLD response that survived correction for multiple comparisons. There were no significant interaction effects between neglect and NP that survived correction for multiple comparisons.

### Potential confounds

The current sample has several potential confounds, including differences in co-morbid psychiatric disorders, age, and IQ. Briefly, we conducted an additional analysis removing individuals with PTSD and an analysis removing individuals with GAD. We also conducted analyses controlling for age, IQ, and scores on the alcohol use disorder identification test (AUDIT) (Fairlie, Sindelar, Eaton, & Spirito, 2006). We also conducted a correlational style analysis using total scores on the neglect and abuse subscales of the CTQ as quantitative covariates (rather than categorical variables), in line with previous work (Blair et al., 2022a, 2022b). We also conducted an analysis covarying for individual learning rates, including an analysis excluding an individual who was an outlier on learning rate. These analyses revealed results similar to the main analysis and are listed in the supplement, with the exception of the analysis where the learning rate outlier was excluded. In this analysis, there was a significant main effect of neglect within rostromedial prefrontal cortex and significant neglect-by-explore interaction effects within dorsolateral prefrontal cortex and superior frontal gyrus.

## Discussion

The aim of the current study was to determine the extent to which neglect and/or abuse was associated with disrupted neural RPE representation in an adolescent sample. We found that within rostromedial prefrontal cortex (rmPFC), ventromedial prefrontal cortex (vmPFC)/anterior cingulate cortex (ACC), individuals with high levels of neglect, relative to those exposed to lower levels of neglect, showed reduced BOLD response modulation by RPE particularly when exploring novel stimuli.

We predicted that ELS, and particularly neglect, would be associated with impaired RPE signaling within medial prefrontal cortex and striatum, particularly when exploring novel stimuli. This prediction was driven by our previous finding that neglect, rather than abuse, was particularly associated with reduced reward responsiveness within rmPFC and striatum during another instrumental learning task (Blair et al., 2022a, 2022b; Hanson et al., 2015). Indeed, RPE signaling was particularly compromised when exploring novel stimuli – previous work has shown that in healthy subjects the RPE signal is particularly *augmented* when responding to novel stimuli (Wittmann et al., 2008). Partially in line with our hypotheses, there were differences in RPE-modulated BOLD response within rmPFC, dlPFC, and premotor cortex between individuals exposed to high levels of neglect relative to individuals exposed to lower levels of neglect. However, contrary to our hypotheses, we did not find an association between neglect exposure and striatal RPE modulation overall. This may reflect issues relating to statistical power and/or that abuse exposure also plays a contributory role (in this regard it is notable that an analysis contrasting individuals who had experienced *any* form of maltreatment relative to TD individuals revealed reduced RPE-modulated BOLD response during explore trials within ventromedial prefrontal cortex, and at trend levels, within striatum; see online Supplemental Material). It should be noted that our results here reflect categorical analyses of abuse and neglect, as recent work has suggested that the test-retest reliability of fMRI data is much lower (Elliott et al., 2020) than would be needed for clinical application or individual-level interpretation (Blair, Mathur, Haines, & Bajaj, 2022b; Chen et al., 2021). However, given our prior work that has used correlational approaches, we have repeated our analyses utilizing a dimensional approach in the Supplement (online Supplementary Table S6). In short, while the current data support suggestions that neglect may particularly compromise reward responsiveness within regions of prefrontal cortex, it is unclear whether this is specifically the case for striatal responsiveness (see also Dillon et al., 2009).

The current study did not show any relationships between abuse and RPE modulation. This finding is consistent with models that describe the relationship between different forms of maltreatment and impacts on neural development (McLaughlin & Sheridan, 2016) and with our previous work (Blair et al., 2022a, 2022b). It has been suggested in the past that different types of childhood ELS (such as abuse *v.* neglect) may have different consequences for neural development. From one theoretical perspective, ELS can be divided into two dimensions: exposure to excessive threat associated with abuse and exposure to excessive deprivation associated with neglect (McLaughlin & Sheridan, 2016). Animal models of ELS have shown that rat pups exposed to excessive threat-related stress showed increased amygdala responses to predator odors (Santiago, Lim, Opendak, Sullivan, & Aoki, 2018) while maternal separations in rat pups was associated with reduced dopamine receptor binding within the basal ganglia and poorer performance on a novel object recognition task (Sinani et al., 2022). Indeed, work from other groups indicates that individuals who have experienced early life abuse show greater amygdala responsiveness to threat (Puetz et al., 2020) while individuals who have experienced early life neglect show reduced striatal responsiveness to reward (Dillon et al., 2009). Consistent with this model, prior work from our group indicate that individuals with histories of abuse show greater responsivity to threatening stimuli within medial and lateral frontal cortex (Blair et al., 2019, 2020) whereas neglect is associated with reduced responsivity to rewarding stimuli within medial frontal cortex and striatum (Blair et al., 2022a, 2022b). However, there has been some empirical work showing alterations in RL associated with prior abuse (Cisler et al., 2019; Letkiewicz et al., 2022). It should be noted, however, that the behavioral paradigms utilized in these studies were substantially different than the one in the current study. In Cisler et al. (2019), an emotional three-armed bandit task using facial stimuli was employed while in Letkiewicz et al. (2022), a fear conditioning paradigm was used. Although both studies compared groups of individuals with and without histories of abuse, neither of these studies report the effects of neglect, so it is difficult to assess whether the effect of childhood abuse was completely isolated. Future work should examine under which specific contexts (such as social referencing, fear conditioning *v.* instrumental learning with monetary values) abuse exposure may be related to impairments in RL. In short, we did not find any evidence of a relationship between abuse and neural representation of RPE.

The current study has several limitations. First, there was a great degree of psychiatric co-morbidity within the sample. It could be argued that the findings in the current study are reflective of psychiatric co-occurrences of neglect rather than neglect itself. Only PTSD and GAD diagnoses were associated with maltreatment group. We therefore conducted sensitivity analyses of the diagnoses that were associated with maltreatment (PTSD and GAD) and obtained similar results, providing partial support that our findings are not confounded by these diagnoses. Moreover, given our prior work on the relationship between RPE-modulated BOLD response and alcohol use disorder symptomatology in an overlapping sample, it could be argued that the findings in the current study are reflective of alcohol use. In our prior work, we found a relationship between AUDIT scores and RPE modulation within striatum, insula, and anterior cingulate cortex (Aloi et al., 2021). When re-running the analysis covarying for scores on the AUDIT, a measure of alcohol use disorder symptomatology, and got a similar result regarding neglect, providing partial support that the current findings are not confounded by alcohol use. We also found that AUDIT scores were related to RPE-modulated BOLD response within striatum and cerebellum, partially replicating our previous result (Aloi et al., 2021). Additionally, given our prior work (Blair et al., 2022a, 2022b), we examined whether RPE-modulated BOLD response within the brain regions identified in the main analysis predicted ADHD or Conduct Problems (see online Supplemental Material). We did not find any associations between RPE-modulated BOLD response and ADHD or conduct problems. It is possible that while these neural differences are related to prior experience of neglect, that there are other differences that may mediate the relationship between neglect and psychopathology. Second, it should be noted that there were no task performance findings related to neglect or abuse in the current study and that there were no significant associations between RPE-modulated BOLD response and task performance. This is in line with our prior work (Blair et al., 2022a, 2022b) which also did not show any significant associations between task performance and neglect or abuse. One possibility is that our current RL paradigms are not sensitive enough to pick up behavioral effects associated with neglect or abuse. A second possibility is that participants with neglect histories are compensating for neural level issues in RPE signaling. Experiences of ELS were assessed through the self-report CTQ and not through any parent/guardian report measures and/or reports to local Child Protective Services. It is therefore possible that some individuals may have experienced neglect and/or abuse that may have not been reported. It should also be noted that regarding our computational modeling approaches, we used an average learning rate in calculating EV's and RPE's and that a logistic regression function was applied to all EV's to calculate NP. This approach may fail to capture more fine-grained individual differences in exploration and exploitation strategies and/or reinforcement learning. Future work should explore these individual differences and whether they may moderate the relationship between abuse and/or neglect and brain activity. Also, this study was a secondary analysis of a larger study examining irritability and conduct problems, and as such, no formal power analysis was conducted prior to running these analyses. As such, these findings should be regarded as preliminary and must be replicated within a study that is designed to investigate the association between ELS and neural dysfunction and designed *a priori* to have sufficient power to detect these effects.

In conclusion, neglect was associated with impairments in RPE signaling within rostromedial/ventromedial prefrontal and lateral frontal cortices during exploration of novel stimuli. The current data support theoretical models (McLaughlin & Sheridan, 2016) and are in line with prior empirical work (Blair et al., 2022a, 2022b) suggesting that childhood ELS, particularly neglect, is associated with impairments in reinforcement processing. However, our prior work only examined unmodulated BOLD responses during a RL task (Blair et al., 2022a, 2022b), whereas the current study examines BOLD responses modulated by RPE; it should be noted that the sample used here does overlap substantially with the prior sample in Blair et al. (2022a, 2022b; *n* = 68 participants overlap). These data extend the findings from Blair et al. (2022a, 2022b) showing that neglect, rather than abuse, might be particularly associated with compromised RPE representation when exploring novel stimuli. Given the association between and RPE signaling and conditions including alcohol use disorder (Blair et al., 2022a, 2022b), disruptive behavior disorders (White et al., 2013), ADHD (Grimm et al., 2021) and depression (Kumar et al., 2018), it will be important to disentangle how impairments in RPE signaling might mediate the relationship between early life neglect and psychopathology in adolescence or adulthood.

## Supporting information

Aloi et al. supplementary material Aloi et al. supplementary material

## Supplementary material

The supplementary material for this article can be found at https://doi.org/10.1017/S0033291724002411.
