# Supplementary material for: Childhood neglect is associated with alterations in neural prediction error signaling and the response to novelty
Source: Psychol Med. 2024 Oct 24;54(14):3930–8. doi: 10.1017/S0033291724002411 (PMC11578899; doi:10.1017/S0033291724002411)
Supplement: Aloi et al. supplementary material [file S0033291724002411sup001.docx]

# ***Main Study: Supplemental Methods***

***Recruitment***

Participants were recruited from a residential youth care facility and the surrounding community. Participants recruited from the residential youth care facility had been referred for behavioral and mental health problems. These procedures are documented in a number of previous studies from our group (Aloi et al., 2021; Blair et al., 2022; Blair et al., 2019). Participants from the community were recruited through flyers and/or social media. Clinical characterization was done through psychiatric interviews by licensed and board-certified child and adolescent psychiatrist with the participants and their caregivers to adhere closely to common clinical practice.

The Boys Town National Research Hospital institutional review board approved this study. A doctoral level researcher or a member of the clinical research team obtained written informed consent and assent. In all cases, youth had the right to decline participation at any time before or during the study.

Participants were recruited from both the residential care facility and the surrounding community. Participants from the residential care facility all had been exposed to high levels of abuse and/or neglect. Participants from the community (n=108) had mostly not been exposed to high levels of abuse or neglect (n=78 out of 108). 10 participants from the community had been exposed to high levels of abuse, but not neglect. 13 participants from the community had been exposed to high levels of neglect, but not abuse. 7 participants from the community had been exposed to high levels of neglect and abuse. CPS reports were made for all participants who reported high levels of abuse and/or neglect.

***Exclusion Criteria***

Exclusion criteria for the broader study included IQ<75 assessed with the Wechsler Abbreviated Scale of Intelligence (WASI) (Wechsler, 2011), pregnancy, non-psychiatric medical conditions that require the use of medication that may have psychotropic effects (e.g., beta blockers, steroids), current psychosis, pervasive developmental disorders, Tourette’s disorder, neurological disorders, presence of metallic objects in the body, and claustrophobia. Current psychiatric conditions (other than psychotic disorders or pervasive developmental disorders) were not exclusionary. Use of psychotropic medications for psychiatric indications (e.g., stimulants, selective serotonin reuptake inhibitors) were not exclusory. However, participants on stimulant medication were asked to withhold medication on the day of scanning.

***Childhood Trauma Questionnaire***

The CTQ is a 28-item self-report measure containing five sub-scale indexing emotional abuse, sexual abuse, physical abuse, emotional neglect, and physical neglect. It has high internal consistency, test-retest reliability, and convergent and discriminant validity with clinical interviews and clinician reports of ELS (Bernstein, Ahluvalia, Pogge, & Handelsman, 1997). Individuals respond to each item on a 5-point Likert scale, with scores ranging from 25 (no reported childhood abuse or neglect) to 125 (very high levels of childhood abuse *and* neglect).

***Novelty Task***

On the Novelty task, participants were instructed to figure out which picture was worth the most money and choose that picture to win the most money. There were no explicit instructions or manipulations regarding the stability or variabilities assigned to each stimulus throughout the task. The task was not rigged to incentivize choosing the novel stimuli. At the beginning of each trial, the three stimuli were presented at one of three randomized locations aligned in a horizontal row. Participants then choose one of the three stimuli via selection on a button box within 1500ms of presentation. If the participant responds within 1500ms, the selected picture is accentuated by a gray border for 1500ms plus a jittered interval between 1000-2000ms. After this interval, the participant receives outcome feedback indicating their winnings for that trial and their overall winnings.

## ***Computational Modeling: Reinforcement Learning Model***

The computational modeling process utilized for this study has been outlined in our previous work (1). Two free parameters of a reinforcement learning model (learning rate, $\alpha$; and inverse temperature, β) were estimated through a nonlinear optimization process by maximizing the likelihood of the actual choices of participants. The probability, *d*, of a choice, *i*, given a value, *v*, was calculated using the softmax rule:

$$P_{i}{dP}_{i}\left( t \right)= \frac{e^{\beta v_{i}(t)}}{\sum_{k=1}^{3} e^{\beta v_{ki}(t)}}P_{i}\left( t \right)= \frac{e^{\beta v_{i}(t)}}{\sum_{k=1}^{3} e^{\beta v_{i}(t)}}$$

where k indexes the three available choices. The log-likelihood was then calculated as follows:

$$ll=-\sum_{t=1}^{T} log\sum_{k=1}^{3} c_{k}(t)d_{k}d(t)$$

Where *c_k_(t)*=1 when the participant chooses option *k* in trial *t* and *c_k_(t)*=0 for all unchosen options per trial. The model, maximizes the choice probability *d_k_(t)* of the actual choices made by participants by minimizing the log- using fminsearch in matlab. The initial values for $\alpha$ were drawn from the standard uniform distribution on the open interval (0,1). The initial values for β were drawn from a standard uniform distribution. The values of the two free parameters were set at the value for which the iteration resulted in the minimum log-likelihood. Based on a larger population of 290 youths who performed the task used here (including current participants), a learning rate of α=0.692 was established and used in the task.

## ***Computational Modeling: Novelty Propensity***

To determine *novelty propensity*, we examined the proportion of times that participants selected the novel stimulus on the *second* trial after the introduction of the novel stimulus. Notably, participants were most likely to pick the novel stimulus on the second trial after its introduction (43.2% versus 33-36% on all other trials after introduction; *t*s=5.77-8.73, *p*s<.001). Participants were not more likely to pick the novel stimulus on the first trial after its introduction relative to the third or more trial after introduction (36.0% as opposed to 33-36%; *t*s=0.24-1.87, *p*s>.05). Participants were most likely to explore the novel stimulus on the second trial after its introduction and we termed these trials “Explore” trials. Participants had a mean of 17.9 trials defined as “Explore”, and a mean of 262.1 trials defined as “Non-Explore.”

To determine an individual’s novelty propensity, a logistic regression function was calculated for each individual; specifically, the EV of the best non-novel option (non-novel stimulus with the highest EV; abbreviated as EV_best_) was used to predict the probability of the participant choosing the novel stimulus on the second trial after a novel stimulus is presented. The novelty propensity for each individual was defined as the EV_best_ for which the participant had a probability of 0.5 of choosing the novel stimulus. In order to ensure that plausible values were calculated, if this calculated value was outside the range of expected values of the best non-novel option for a participant, then it was set to either the minimum EV_best_ (if less than the minimum; N=2) or the maximum EV_best_ (if greater than the maximum; N=13). Based on a larger population of 290 youths who performed the task (including current participants), the average novelty propensity was 0.216. In other words, the average participant had an EV of 0.216 for the best alternative on trials where they had a 50% likelihood of selecting the novel stimulus. Therefore, novel stimuli were assigned an EV of 0.216 on the first time they were selected.

***Functional MRI Parameters***

Whole-brain functional MRI data were acquired via 3T MAGNETOM Skyra magnetic resonance imaging scanner (Siemens medical solutions). The total amount of time for each run of the task varied slightly based on reaction time. 242-275 functional images were taken for each run with a T2* weighted gradient echo planar imaging (EPI) sequence (repetition time=2500ms; echo time=27ms; 240mm field of view; 94x94 matrix; 90^o^ flip angle). Whole-brain coverage for each run was obtained with 43 axial slices (thickness=2.5mm; voxel size=2.6x2.6x2.5mm^3^). A high-resolution T1 anatomical scan (MPRAGE, repetition time =2200ms, echo time=2.48ms; 230mm field of view; 8^o^ flip angle; 256x208 matrix, thickness=1mm; voxel size=0.9x0.9x1mm^3^) was obtained in register with the EPI dataset. Whole-brain coverage was obtained with 176 axial slices.

***Functional MRI Analysis***

Functional MRI data were analyzed using Analysis of Functional NeuroImages (AFNI) (Cox, 1996). First, the first four volumes collected prior to magnetization were discarded. Then, each anatomical scan for each participant was registered to the Talairach and Tournoux atlas (Talairach & Tournoux, 1988) and each participant’s EPI data were registered to their Talairach anatomical scan. Functional images were motion corrected to the initial volume of the first functional run and spatially smoothed with a 6mm full-width at half-maximum Gaussian kernel. The EPI data then underwent time-series normalization to a T1-weighted image, and these results were multiplied by 100 for each voxel. The resultant regression coefficients are representative of a percentage of signal change from the mean.

Data were analyzed with a random-effects general linear model (GLM) within AFNI. Six task regressors were generated: 1) cue phase on non-explore trials, 2) cue phase on explore trials, 3) feedback phase on non-explore trials, 4) feedback phase on explore trials, 5) cue phase on trials where participants did not respond within the 1500ms window, and 6) feedback phase on trials where participants did not respond within the 1500ms window. BOLD response amplitude was parametrically modulated by EV at each voxel/time point for the cue phase and by RPE at each voxel/time point for the feedback phase. Each volume and its predecessor on which motion exceeded 0.5mm was censored. GLM fitting was performed with six task regressors, six motion regressors, and a regressor modeling a baseline drift function. Volumes exceeding 0.5mm motion were censored via removal from the time series. This procedure generated unmodulated β-coefficients/*t*-statistics for each voxel and regressor. EV-modulated β-coefficients/*t*-statistics for each voxel and regressor were generated for the cue phase. RPE-modulated β-coefficients/*t*-statistics for each voxel and regressor for each voxel and regressor were generated for the feedback phase.

***Multiple Comparison Correction***

All clusters were clusterwise corrected to *p*<.05 using a spatial clustering operation in AFNI’s 3dClustSim utilizing the autocorrelation function (-acf) with 10,000 Monte Carlo simulations for the whole-brain analysis. Spatial autocorrelation was estimated using the residuals from the individual-level GLM analyses. The initial voxelwise threshold was set at *p*=.001 (Cox, Chen, Glen, Reynolds, & Taylor, 2017). This procedure yielded and extant threshold of *k*=17 contiguous voxels for the whole-brain analysis (NN1/facewise neighbor clustering).

***Prediction of Conduct Problems and ADHD Symptoms***

Our prior work (Blair et al., 2022) showed that dysfunction within striatum and medial prefrontal cortex predicted conduct problems and ADHD symptoms. We ran linear regressions using RPE-modulated BOLD response during Novel and Non-novel trials within our four clusters showing significant neglect-by-explore interactions (ACC/vmPFC/rmPFC, dlPFC, postcentral gyrus, and superior frontal gyrus) to predict conduct problems on the Strength and Difficulties Questionnaire (Goodman, 1997) (N=166 with available SDQ data) and Conners ADHD symptoms (N=178 with available Conners data) (Conners, 2008). The independent variables included in each of the regression models were Novelty Propensity, RPE-modulated BOLD response during non-explore trials, and RPE-modulated BOLD response during explore trials. These analyses were run in SPSS.

# ***Main Study: Supplemental Results***

## ***Excluded Subjects***

53 participants were excluded from the fMRI analysis due to excessive movement, fMRI artifact, or missing questionnaire data. The average age of these 53 participants was 14.1 years old (SD=2.60), the average IQ was 102.3 (SD=15.71), and was 62.8% male. The had an average score on the CTQ Emotional Abuse subscale of 7.8 (SD=4.01), Physical Abuse subscale of 6.6 (SD=3.11), Sexual Abuse subscale of 6.7 (SD=4.40), Emotional Neglect subscale of 7.7 (SD=3.67) and Physical Neglect subscale of 7.6 (SD=4.03). There were no differences between the excluded participants and the included participants on age, IQ, or CTQ subscales or total scores (*t*s<1.93, *p*s>.05). There were no differences in gender distribution between the excluded participants and the included participants (χ^2^=1.10, *p*>.05).

## ***Main Effects***

Brain regions showing significant main effects of RPE modulation can be found in Table S1.

## ***Potential Confounds***

Our sample has several potential confounds, including co-morbid psychiatric disorders, age, and IQ. Post-traumatic Stress Disorder (PTSD) and Generalized Anxiety Disorder (GAD) diagnoses were both significantly associated with greater percentages of individuals in the co-morbid abuse and neglect groups. Therefore, we ran two additional ANOVAs that mirrored our main analysis, except one ANOVA excluded participants with PTSD and another ANOVA excluded participants with GAD.

The average learning rate for the larger sample from which the sample for the current study comes from is 0.692. The average learning rate for the current sample is 0.89 (SE=0.194). However, this was due to the presence of one individual who was an outlier. Excluding this individual, the average learning rate for the sample in the current study is 0.70 (SE=0.028). We re-ran our analysis excluding this participant. We also re-ran our analysis covarying for learning rate.

In analyses where additional multiple participants were excluded, we used an initial threshold of *p*<.005 with a minimum cluster size of *k*=40 voxels. We used a less stringent threshold because our primary goal in these supplemental analyses was to show that the reported effects are robust to these analyses, so we placed an emphasis on sensitivity to detect effects. We have noted in the supplemental tables which effects survive at the original threshold of *p*<.001 with a minimum cluster size of *k*=16 voxels.

***PTSD***

When excluding participants with PTSD, the significant main effect of neglect within ventromedial/rostromedial prefrontal cortex was maintained from the main analysis. The significant neglect-by-explore interaction within ventromedial/rostromedial prefrontal cortex, dorsolateral prefrontal cortex, and postcentral gyrus, were maintained from the main analysis. For full results, see Table S2.

***GAD***

When excluding participants with GAD, the significant main effect of neglect within ventromedial/rostromedial prefrontal cortex result was maintained from the main analysis. The significant neglect-by-explore interaction within rostromedial prefrontal cortex and postcentral gyrus results were maintained from the main analysis. For full results, see Table S3.

***IQ***

Moreover, group was significantly associated with age and IQ. Therefore, we ran two additional ANCOVAs that mirrored our main analysis, controlling for age and IQ.

The significant main effect of neglect was maintained within anterior cingulate/ventromedial prefrontal cortex and rostromedial prefrontal cortex when controlling for age. The significant neglect-by-explore was maintained within dorsolateral prefrontal cortex, postcentral gyrus, superior frontal gyrus, and, at trend levels, anterior cingulate and dorsolateral prefrontal cortex when controlling for age. For both analyses, the Abuse-by-Neglect-by-Explore interaction within cuneus was also maintained. For full results see Table S4.

When controlling for IQ, the significant main effect of neglect was maintained within anterior cingulate/ventromedial prefrontal cortex and rostromedial prefrontal cortex. The significant neglect-by-explore interaction effect was maintained within dorsolateral prefrontal cortex, rostromedial prefrontal cortex, anterior cingulate cortex, and superior frontal gyrus. For full results see Table S5.

***Dimensional (Individual Differences) Analysis***

We also ran a dimensional analysis examining the effects of neglect and abuse as continuous variables on BOLD responses modulated by RPE. In this analysis the ANCOVA mirrored our main analysis, except instead of using groups as between-subject variables, we used scores on the CTQ abuse and neglect subscales as between-subject covariates. The significant main effect of neglect in anterior cingulate/ventromedial prefrontal cortex and dorsolateral prefrontal cortex was maintained from the main analysis. The significant neglect-by-explore interaction effect within dorsolateral prefrontal cortex was maintained from the main analysis. For full results see Table S6.

***Alcohol Use Disorder (AUD) Symptoms***

Given our prior work on the relationship between AUD symptomatology and RPE-modulation on the Novelty task (1), we also re-ran our analyses covarying for AUDIT scores. The significant main effect of neglect in rostromedial prefrontal cortex and dorsolateral prefrontal cortex was maintained from the main analysis. The significant neglect-by-explore interaction effect within rostromedial prefrontal cortex, dorsolateral prefrontal cortex, superior frontal gyrus, and postcentral gyrus were maintained from the main analysis. For full results see Table S7.

In all instances the results of these supplemental analyses were highly similar to the main analysis in the manuscript.

***Main Effect of Maltreatment***

We ran a supplemental analysis in which we compared individuals with *any* history of ELS (i.e., abuse, neglect, or co-morbid abuse and neglect) to the HC group. In this analysis there was a significant main effect of CTQ on RPE modulation within caudate, ventromedial prefrontal cortex, and precentral gyrus, such that individuals in the maltreatment group showed reduced BOLD response modulated by RPE. There was a significant CTQ-by-Explore interaction effect within insula and ventromedial prefrontal cortex. For full results, see Table S8.

***Cue Phase Data***

For completeness, we ran a supplemental analysis in which we conducted a 2 (Neglect: High, Low)x2 (Abuse: High, Low) repeated measures ANOVA on cue-phase BOLD response data modulated by EV. We could not run an analysis incorporating the Explore trials because the EV would have been the same on all Explore trials (0.216), resulting in multicollinearity. We found that there were significant abuse-by-neglect interactions within middle temporal gyrus and inferior parietal cortex. See Table S9 for further details.

***Exclusion of Learning Rate Outlier***

We ran a supplemental analysis in which we excluded one individual who was an outlier on the learning rate. The rostromedial prefrontal cortex finding was maintained from the main analysis for the main effect of neglect. The dorsolateral prefrontal cortex finding was maintained from the main analysis for the neglect-by-explore interaction effect. See Table S10 for further details.

***Corvarying for Learning Rate***

We ran a supplemental analysis in which we covaried for individual learning rates. The rostromedial prefrontal cortex was maintained from the main analysis for the main effect of neglect. The dorsolateral prefrontal cortex finding was maintained from the main analysis for the neglect-by-explore interaction effect. See Table S11 for further details.

***Cue Phase Data Covarying for Learning Rate***

We ran a supplemental analysis in which we conducted a 2 (Neglect: High, Low)x2 (Abuse: High, Low) repeated measures ACNOVA on cue-phase BOLD response data modulated by EV with individual learning rate as a covariate. We found that there were significant abuse-by-neglect interactions within middle temporal gyrus and inferior parietal cortex. See Table S12 for further details.

***Prediction of Conduct Problems and ADHD Symptoms***

Our regression models did not significantly predict SDQ conduct problems (Fs<2.34, *p*s>.05) or ADHD symptoms (Fs<1.67, *p*s>.05).

**Supplemental References**

Aloi, J., Crum, K. I., Blair, K. S., Zhang, R., Bashford-Largo, J., Bajaj, S., . . . Blair, R. J. R. (2021). Individual associations of adolescent alcohol use disorder versus cannabis use disorder symptoms in neural prediction error signaling and the response to novelty. *Developmental Cognitive Neuroscience*, 48, 100944. doi:10.1016/j.dcn.2021.100944

Bernstein, D. P., Ahluvalia, T., Pogge, D., & Handelsman, L. (1997). Validity of the childhood trauma questionnaire in an adolescent psychiatric population. *Journal of the American Academy of Child and Adolescent Psychiatry*, 36(3), 340-348. doi:https://doi.org/10.1097/00004583-199703000-00012

Blair, K. S., Aloi, J., Bashford-Largo, J., Zhang, R., Elowsky, J., Lukoff, J., . . . Blair, R. J. (2022). Different forms of childhood maltreatment have different impacts on the neural systems involved in the representation of reinforcement value. *Developmental Cognitive Neuroscience*, 53, 101051. doi:10.1016/j.dcn.2021.101051

Blair, K. S., Aloi, J., Crum, K., Meffert, H., White, S. F., Taylor, B. K., . . . Blair, R. J. (2019). Association of Different Types of Childhood Maltreatment With Emotional Responding and Response Control Among Youths. *JAMA Network Open*, 2(5), e194604. doi:10.1001/jamanetworkopen.2019.4604

Conners, C. (2008). *Conners 3rd Edition Manual*. Toronto, Ontario, Canada: Multi-Health Systems.

Cox, R. W. (1996). AFNI: Software for analysis and visualization of functional magnetic resonance neuroimages. *Computers and Biomedical Research*, 29(3), 162-173. doi:10.1006/cbmr.1996.0014

Cox, R. W., Chen, G., Glen, D. R., Reynolds, R. C., & Taylor, P. A. (2017). fMRI clustering and false-positive rates. *Proceedings of the National Academy of Sciences*, 114(17), E3370-E3371. doi:10.1073/pnas.1614961114

Goodman, R. (1997). The Strengths and Difficulties Questionnaire: a research note. *Journal of Child Psychology and Psychiatry*, 38(5), 581-586. doi:10.1111/j.1469-7610.1997.tb01545.x

Talairach, J., & Tournoux, P. (1988). *Co-Planar Stereotaxis Atlas of the Human Brain: an approach to cerebral imaging*. (Vol. 270).

Wechsler, D. (2011). *Wechsler Abbreviated Scale of Intelligence-Second Edition*. San Antonio, TX: NCS Pearson.

# ***Supplemental Tables***

| Table S1. Brain regions demonstrating main effects of RPE modulation | | | | | | | | |
| --- | --- | --- | --- | --- | --- | --- | --- | --- |
| Coordinates of Peak Activation^b^ | | | | | | | | |
| Main effects of RPE modulation (Grand mean of Novel and Non-novel trials) | | | | | | | | |
| Region^a^ | Hemisphere | BA | x | y | z | *F*(1,174) | Partial η^2^ | Voxels |
| Ventral Striatum | R | - | 11 | 5 | -4 | 34.28 | 0.168 | 41 |
| Posterior Cingulate Cortex | L | 31 | -4 | -40 | 38 | 17.72 | 0.094 | 31 |
| Ventromedial Prefrontal Cortex | L | 32 | -10 | 41 | -1 | 19.10 | 0.101 | 28 |
| Inferior Parietal Lobule | L | 40 | -43 | -55 | 47 | 17.23 | 0.092 | 20 |
| Inferior Parietal Lobule | L | 40 | -43 | -31 | 29 | 24.56 | 0.126 | 18 |
| Main effect of RPE modulation during explore trials | | | | | | | | |
| Middle Temporal Gyrus | L | 21 | -62 | -38 | 0 | 31.44 | 0.151 | 82 |
| Precuneus | L | 31 | -4 | -40 | 38 | 18.53 | 0.095 | 29 |
| Middle Temporal Gyrus | L | 37 | -50 | -50 | -6 | 18.44 | 0.095 | 18 |
| Anterior Cingulate Cortex^c^ | R/L | 24 | 4 | 28 | 18 | 18.11 | 0.093 | 10 |
| Caudate^c^ | L | - | -2 | 10 | 12 | 17.41 | 0.090 | 12 |
| Main effect of RPE modulation during non-explore trials | | | | | | | | |
| Ventral Striatum/Dorsal Striatum/Medial Prefrontal Cortex/Anterior Cingulate Cortex | L | - | -14 | 8 | -4 | 91.97 | 0.342 | 3122 |
| Posterior Cingulate Cortex | L | 30 | -8 | 52 | 14 | 55.16 | 0.238 | 720 |
| Postcentral Gyrus | R | 2 | 50 | -22 | 44 | 35.44 | 0.167 | 311 |
| Cingulate Gyrus | L | 32 | -10 | 16 | 38 | 33.52 | 0.159 | 282 |
| Middle Temporal Gyrus | L | 37 | -58 | -46 | -4 | 44.00 | 0.199 | 110 |
| Precuneus | R | 7 | 4 | -62 | 42 | 25.40 | 0.125 | 108 |
| Postcentral Gyrus | R | 3 | 28 | -34 | 50 | 25.55 | 0.126 | 71 |
| Insula | R | 13 | 32 | 20 | 14 | 25.14 | 0.124 | 47 |
| Middle Frontal Gyrus | R | 6 | 34 | -4 | 50 | 19.82 | 0.101 | 45 |
| Superior Temporal Gyrus | R | 13/40 | 50 | -46 | 20 | 22.28 | 0.112 | 44 |
| Claustrum | L | 13 | -28 | 16 | 18 | 25.70 | 0.127 | 37 |
| Precentral Gyrus | R | 9 | 40 | 14 | 36 | 19.01 | 0.097 | 37 |
| Superior Parietal Lobule | R | 5 | -22 | -44 | 62 | 23.03 | 0.115 | 34 |
| Precentral Gyrus | R | 6 | 56 | -2 | 8 | 26.44 | 0.130 | 31 |
| Cerebellar Tonsil | R | - | 32 | -56 | -40 | 24.52 | 0.122 | 29 |
| Middle Temporal Gyrus | R | 39 | 44 | -74 | 26 | 26.41 | 0.130 | 25 |
| Culmen | L | - | -4 | -38 | -18 | 30.79 | 0.148 | 24 |
| Culmen | L | 20 | -32 | -38 | -18 | 22.31 | 0.112 | 19 |
| Precentral Gyrus | L | 6 | -26 | -10 | 50 | 13.63 | 0.071 | 18 |

Note: ^a^ According to the Talairach Daemon Atlas (<http://www.nitrc.org/projects/tal-daemon/>), ^b^ Based on
the Tournoux & Talairach standard brain template, ^c^Below ClustSim generated threshold, BA= Brodmann’s Area

| Table S2. Brain regions demonstrating significant Neglect Effects and Neglect-by-Explore Effects removing individuals with PTSD (corrected at initial p<.005) | | | | | | | | |
| --- | --- | --- | --- | --- | --- | --- | --- | --- |
| Coordinates of Peak Activation^b^ | | | | | | | | |
| Region^a^ | Hemisphere | BA | x | y | z | *F*(1,154) | Partial η^2^ | Voxels |
| Main Effect of Neglect | | | | | | | | |
| Ventromedial/Rostromedial Prefrontal Cortex* | L | 32 | 5 | 41 | 11 | 18.38 | 0.098 | 139 |
| Insula^ | R | 13 | 41 | 2 | 11 | 17.39 | 0.093 | 85 |
| Insula^ | L | 13 | -37 | 8 | 8 | 13.82 | 0.075 | 79 |
| Postcentral Gyrus | R | 3 | -13 | -37 | 59 | 14.88 | 0.080 | 73 |
| Neglect-by-Explore | | | | | | | | |
| Ventromedial/Rostromedial Prefrontal Cortex* | R | 9/10/32 | 5 | 44 | 14 | 18.46 | 0.098 | 46 |
| Dorsolateral prefrontal cortex* | R | 10 | 29 | 59 | 2 | 23.89 | 0.102 | 45 |
| Dorsolateral prefrontal cortex* | L | 10 | -25 | 59 | 17 | 20.16 | 0.106 | 41 |
| Postcentral Gyrus*^ | L | 3 | -22 | -28 | 53 | 14.07 | 0.076 | 121 |
| Precuneus | L/R | 5 | 11 | -34 | 53 | 12.93 | 0.071 | 42 |

Note: ^a^ According to the Talairach Daemon Atlas (<http://www.nitrc.org/projects/tal-daemon/>), ^b^ Based on
the Tournoux & Talairach standard brain template, ^c^ Below the ClustSim established threshold, BA= Brodmann’s Area,
* Overlapping or proximal to a significant cluster in the main analysis, ^ survives at a threshold of *p*<.001 and *k*=16 voxels

| Table S3. Brain regions demonstrating significant Neglect Effects and Neglect-by-Explore Effects removing individuals with GAD (initial p<.005) | | | | | | | | |
| --- | --- | --- | --- | --- | --- | --- | --- | --- |
| Coordinates of Peak Activation^b^ | | | | | | | | |
| Region^a^ | Hemisphere | BA | x | y | z | *F*(1,135) | Partial η^2^ | Voxels |
| Main Effect of Neglect | | | | | | | | |
| Ventromedial/Rostromedial Prefrontal Cortex*^ | R/L | 9/32 | 5 | 41 | 14 | 16.78 | 0.098 | 178 |
| Dorsomedial Prefrontal Cortex^ | R | 6 | 14 | -4 | 53 | 23.48 | 0.132 | 59 |
| Inferior Frontal Gyrus^ | R | 9 | 50 | -1 | 23 | 11.96 | 0.141 | 255 |
| Insula^ | L | 13 | -34 | 8 | 11 | 15.01 | 0.089 | 191 |
| Postcentral Gyrus^ | R | 40 | 23 | -37 | 53 | 21.76 | 0.113 | 294 |
| Postcentral Gyrus | L | 3 | -13 | -37 | 59 | 20.83 | 0.119 | 251 |
| Neglect-by-Explore | | | | | | | | |
| Rostromedial Prefrontal Cortex* | R | 9 | 2 | 53 | 35 | 17.24 | 0.101 | 90 |
| Precentral Gyrus | R | 4 | 32 | -22 | 53 | 11.97 | 0.072 | 55 |
| Postcentral Gyrus*^ | L | 3 | -22 | -28 | 53 | 17.37 | 0.101 | 270 |
| Inferior Parietal Lobule | R | 40 | 38 | -43 | 47 | 13.81 | 0.082 | 42 |
| Insula^ | L | 13 | -31 | 5 | 20 | 18.00 | 0.105 | 80 |
| Insula | R | 13 | 44 | 8 | 17 | 14.05 | 0.084 | 69 |

Note: ^a^ According to the Talairach Daemon Atlas (<http://www.nitrc.org/projects/tal-daemon/>), ^b^ Based on
the Tournoux & Talairach standard brain template, ^c^ Below the ClustSim established threshold, BA= Brodmann’s Area,
*Overlapping with or proximal to a cluster reported in the main analysis, ^ survives at a threshold of *p*<.001 and *k*=16 voxels

| Table S4. Brain regions demonstrating significant Neglect Effects and Neglect-by-Explore Effects Controlling for Age | | | | | | | | |
| --- | --- | --- | --- | --- | --- | --- | --- | --- |
| Coordinates of Peak Activation^b^ | | | | | | | | |
| Region^a^ | Hemisphere | BA | x | y | z | *F*(1,173) | Partial η^2^ | Voxels |
| Main Effect of Neglect | | | | | | | | |
| Anterior Cingulate Cortex/Ventromedial Prefrontal Cortex* | R | 32 | 5 | 41 | 11 | 18.34 | 0.098 | 20 |
| Rostromedial Prefrontal Cortex* | R | 9 | 8 | 44 | 29 | 17.95 | 0.096 | 18 |
| Neglect-by-Explore | | | | | | | | |
| Dorsolateral Prefrontal Cortex* | R | 10 | 29 | 56 | 20 | 18.51 | 0.099 | 20 |
| Superior Frontal Gyrus | R | 6 | 20 | 14 | 56 | 17.24 | 0.093 | 20 |
| Anterior Cingulate Cortex*^c^ | R | 9 | 5 | 44 | 14 | 18.36 | 0.098 | 13 |
| Dorsolateral Prefrontal Cortex*^c^ | R | 10 | 29 | 59 | 2 | 19.04 | 0.101 | 11 |
| Postcentral Gyrus* | L | 3 | -22 | -31 | 53 | 18.21 | 0.097 | 20 |
| Abuse-by-Neglect-by-NP-by-Explore Interaction | | | | | | | | |
| Cuneus | R | 19 | 29 | -82 | 26 | 19.64 | 0.104 | 20 |

Note: ^a^ According to the Talairach Daemon Atlas (<http://www.nitrc.org/projects/tal-daemon/>), ^b^ Based on
the Tournoux & Talairach standard brain template, ^c^ Below the ClustSim established threshold, BA= Brodmann’s Area,
* Overlapping with or proximal to a cluster reported in the main analysis

| Table S5. Brain regions demonstrating significant Neglect Effects and Neglect-by-Explore Effects Controlling for IQ | | | | | | | | |
| --- | --- | --- | --- | --- | --- | --- | --- | --- |
| Coordinates of Peak Activation^b^ | | | | | | | | |
| Region^a^ | Hemisphere | BA | x | y | z | *F*(1,173) | Partial η^2^ | Voxels |
| Main Effect of Neglect | | | | | | | | |
| Rostromedial Prefrontal Cortex* | R | 32 | 5 | 41 | 11 | 17.60 | 0.095 | 17 |
| Anterior Cingulate Cortex/Ventromedial Prefrontal Cortex*^c^ | R | 9 | 8 | 44 | 29 | 18.60 | 0.100 | 13 |
| Neglect-by-Explore | | | | | | | | |
| Dorsolateral Prefrontal Cortex* | R | 10 | 29 | 59 | 2 | 24.87 | 0.129 | 31 |
| Rostromedial Prefrontal Cortex/Anterior Cingulate Cortex* | R | 9 | 5 | 44 | 14 | 19.82 | 0.106 | 18 |
| Superior Frontal Gyrus | R | 6 | 20 | 14 | 56 | 17.12 | 0.092 | 17 |

Note: ^a^ According to the Talairach Daemon Atlas (<http://www.nitrc.org/projects/tal-daemon/>), ^b^ Based on
the Tournoux & Talairach standard brain template, ^c^ Below the ClustSim established threshold, BA= Brodmann’s Area,
*Overlapping with or proximal to a cluster reported in the main analysis

| Table S6. Brain regions demonstrating significant Neglect Effects and Neglect-by-Explore Effects (with Neglect and Abuse as Continuous Covariates) | | | | | | | | |
| --- | --- | --- | --- | --- | --- | --- | --- | --- |
| Coordinates of Peak Activation^b^ | | | | | | | | |
| Region^a^ | Hemisphere | BA | x | y | z | *F*(1,174) | Partial η^2^ | Voxels |
| Main Effect of Neglect | | | | | | | | |
| Anterior Cingulate/Ventromedial Prefrontal Cortex* | R | 32 | 5 | 41 | 11 | 20.81 | 0.108 | 41 |
| Dorsolateral Prefrontal Cortex | L | 10 | -22 | 56 | 20 | 22.45 | 0.116 | 36 |
| Precentral Gyrus | L | 4 | -13 | -31 | 65 | 19.23 | 0.101 | 33 |
| Paracentral Lobule | R | 5 | 14 | -34 | 50 | 21.59 | 0.112 | 29 |
| Neglect-by-Explore | | | | | | | | |
| Dorsolateral Prefrontal Cortex* | R | 10 | -22 | 59 | 20 | 21.45 | 0.111 | 42 |
| Precentral Gyrus | L | 4 | -13 | -28 | 65 | 19.53 | 0.103 | 41 |
| Paracentral Lobule | R | 5 | 14 | -34 | 50 | 19.19 | 0.101 | 23 |

Note: ^a^ According to the Talairach Daemon Atlas (<http://www.nitrc.org/projects/tal-daemon/>), ^b^ Based on
the Tournoux & Talairach standard brain template, ^c^ Below the ClustSim established threshold, BA= Brodmann’s Area,
*Overlapping with or proximal to a cluster reported in the main analysis

| Table S7. Brain regions demonstrating significant Neglect Effects and Neglect-by-Explore Effects Controlling for AUDIT scores (initial p<.005) | | | | | | | | |
| --- | --- | --- | --- | --- | --- | --- | --- | --- |
| Coordinates of Peak Activation^b^ | | | | | | | | |
| Region^a^ | Hemisphere | BA | x | y | z | *F*(1,173) | Partial η^2^ | Voxels |
| Main Effect of Neglect | | | | | | | | |
| Rostromedial Prefrontal Cortex* | R | 10 | 20 | 59 | 23 | 21.41 | 0.141 | 59 |
| Superior Frontal Gyrus | R | 6 | 20 | 14 | 56 | 15.49 | 0.106 | 46 |
| Dorsolateral Prefrontal Cortex | L | 10 | -28 | 44 | 26 |  |  | 40 |
| Postcentral Gyrus | L/R | 3/4 | -13 | -37 | 62 | 13.75 | 0.096 | 168 |
| Neglect-by-Explore | | | | | | | | |
| Dorsolateral Prefrontal Cortex | L | 10 | -25 | 59 | 17 | 15.74 | 0.108 | 95 |
| Rostromedial Prefrontal Cortex* | R | 9 | 5 | 56 | 29 | 19.64 | 0.131 | 89 |
| Superior Frontal Gyrus | R | 6 | 20 | 14 | 56 | 13.68 | 0.095 | 51 |
| Postcentral Gyrus | L/R | 3/4 | -13 | -37 | 62 | 12.66 | 0.089 | 149 |
| Main Effect of AUDIT | | | | | | | | |
| Cerebellum | L/R | - | -4 | -43 | -22 | 29.47 | 0.159 | 148 |
| Ventral Putamen/Anterior Insula | R | - | 23 | 11 | -4 | 16.75 | 0.097 | 90 |
| Cerebellum | L | - | -34 | -49 | -25 | 20.96 | 0.118 | 58 |
| Caudate | L | - | -13 | 14 | 8 | 17.63 | 0.102 | 54 |
| Thalamus | R | - | 8 | -19 | 2 | 25.68 | 0.141 | 45 |
| AUDIT-by-Explore | | | | | | | | |
| Cerebellum | L/R | - | -4 | -46 | -22 | 32.77 | 0.174 | 135 |
| Caudate | L | - | -7 | 11 | 11 | 18.15 | 0.104 | 45 |
| Thalamus | R | - | 8 | -19 | 2 | 17.72 | 0.102 | 40 |

Note: ^a^ According to the Talairach Daemon Atlas (<http://www.nitrc.org/projects/tal-daemon/>), ^b^ Based on
the Tournoux & Talairach standard brain template, ^c^ Below the ClustSim established threshold, BA= Brodmann’s Area,
*Overlapping with or proximal to a cluster reported in the main analysis

| Table S8. Brain regions demonstrating significant Effects of ELS | | | | | | | | |
| --- | --- | --- | --- | --- | --- | --- | --- | --- |
| Coordinates of Peak Activation^b^ | | | | | | | | |
| Region^a^ | Hemisphere | BA | x | y | z | *F*(1,174) | Partial η^2^ | Voxels |
| Main Effect of ELS | | | | | | | | |
| Anterior Cingulate Cortex/Ventromedial Prefrontal Cortex | L | 24 | -7 | 35 | 5 | 19.07 | 0.099 | 32 |
| Caudate | L | - | -13 | 5 | 8 | 21.98 | 0.112 | 18 |
| Precentral Gyrus | R | 4 | 17 | -31 | 59 | 19.14 | 0.099 | 43 |
| ELS-by-Explore | | | | | | | | |
| Anterior Cingulate Cortex/Ventromedial Prefrontal Cortex | L | 24 | -4 | 26 | -1 | 23.42 | 0.119 | 20 |
| Insula | R | 13 | 38 | -7 | 14 | 20.94 | 0.107 | 27 |

Note: ^a^ According to the Talairach Daemon Atlas (<http://www.nitrc.org/projects/tal-daemon/>), ^b^ Based on
the Tournoux & Talairach standard brain template, ^c^ Below the ClustSim established threshold, BA= Brodmann’s Area,
*Overlapping with or proximal to a cluster reported in the main analysis

| Table S9. Brain regions demonstrating significant effects on EV-modulated BOLD response | | | | | | | | |
| --- | --- | --- | --- | --- | --- | --- | --- | --- |
| Coordinates of Peak Activation^b^ | | | | | | | | |
| Region^a^ | Hemisphere | BA | x | y | z | *F*(1,174) | Partial η^2^ | Voxels |
| Abuse-by-Neglect Interaction Effects | | | | | | | | |
| Middle Temporal Gyrus | R | 39 | 47 | -70 | 14 | 23.89 | 0.121 | 33 |
| Inferior Parietal Lobule | L | 40 | -55 | -31 | 35 | 18.45 | 0.096 | 22 |
| Middle Temporal Gyrus | L | 22/39 | -55 | -52 | 5 | 18.71 | 0.097 | 18 |

Note: ^a^ According to the Talairach Daemon Atlas (<http://www.nitrc.org/projects/tal-daemon/>), ^b^ Based on
the Tournoux & Talairach standard brain template, ^c^ Below the ClustSim established threshold, BA= Brodmann’s Area,
*Overlapping with or proximal to a cluster reported in the main analysis

| Table S10. Brain regions demonstrating significant Neglect Effects and Neglect-by-Explore Effects Excluding Learning Rate Outlier | | | | | | | | |
| --- | --- | --- | --- | --- | --- | --- | --- | --- |
| Coordinates of Peak Activation^b^ | | | | | | | | |
| Region^a^ | Hemisphere | BA | x | y | z | *F*(1,170) | Partial η^2^ | Voxels |
| Main Effect of Neglect | | | | | | | | |
| Rostromedial Prefrontal Cortex* | R | 32 | 8 | 44 | 29 | 18.48 | 0.098 | 23 |
| Neglect-by-Explore | | | | | | | | |
| Dorsolateral Prefrontal Cortex* | R | 10 | 29 | 59 | 2 | 25.79 | 0.132 | 36 |
| Superior Frontal Gyrus | R | 6 | 20 | 14 | 56 | 18.07 | 0.097 | 17 |

Note: ^a^ According to the Talairach Daemon Atlas (<http://www.nitrc.org/projects/tal-daemon/>), ^b^ Based on
the Tournoux & Talairach standard brain template, ^c^ Below the ClustSim established threshold, BA= Brodmann’s Area,
*Overlapping with or proximal to a cluster reported in the main analysis

| Table S11. Brain regions demonstrating significant Neglect Effects and Neglect-by-Explore Effects with Learning Rate as a Covariate | | | | | | | | |
| --- | --- | --- | --- | --- | --- | --- | --- | --- |
| Coordinates of Peak Activation^b^ | | | | | | | | |
| Region^a^ | Hemisphere | BA | x | y | z | *F*(1,172) | Partial η^2^ | Voxels |
| Main Effect of Neglect | | | | | | | | |
| Rostromedial Prefrontal Cortex* | R | 32 | 8 | 44 | 29 | 17.07 | 0.092 | 21 |
| Neglect-by-Explore | | | | | | | | |
| Dorsolateral Prefrontal Cortex* | R | 10 | 32 | 56 | 17 | 17.93 | 0.094 | 25 |
| Superior Frontal Gyrus | R | 6 | 20 | 14 | 56 | 18.13 | 0.097 | 21 |

Note: ^a^ According to the Talairach Daemon Atlas (<http://www.nitrc.org/projects/tal-daemon/>), ^b^ Based on
the Tournoux & Talairach standard brain template, ^c^ Below the ClustSim established threshold, BA= Brodmann’s Area,
*Overlapping with or proximal to a cluster reported in the main analysis

| Table S12. Brain regions demonstrating significant effects on EV-modulated BOLD response covarying for Learning Rate | | | | | | | | |
| --- | --- | --- | --- | --- | --- | --- | --- | --- |
| Coordinates of Peak Activation^b^ | | | | | | | | |
| Region^a^ | Hemisphere | BA | x | y | z | *F*(1,174) | Partial η^2^ | Voxels |
| Abuse-by-Neglect Interaction Effects | | | | | | | | |
| Middle Temporal Gyrus | R | 39 | 47 | -70 | 14 | 24.02 | 0.124 | 31 |
| Inferior Parietal Lobule | L | 40 | -55 | -31 | 35 | 18.14 | 0.097 | 18 |
| Middle Temporal Gyrus | L | 22/39 | -55 | -52 | 5 | 18.84 | 0.100 | 18 |

Note: ^a^ According to the Talairach Daemon Atlas (<http://www.nitrc.org/projects/tal-daemon/>), ^b^ Based on
the Tournoux & Talairach standard brain template, ^c^ Below the ClustSim established threshold, BA= Brodmann’s Area,
*Overlapping with or proximal to a cluster reported in the main analysis
